# High systemic levels of interleukin-10, interleukin-22 and C-reactive protein in Indian patients are associated with low *in vitro *replication of HIV-1 subtype C viruses

**DOI:** 10.1186/1742-4690-7-15

**Published:** 2010-03-09

**Authors:** Juan F Arias, Reiko Nishihara, Manju Bala, Kazuyoshi Ikuta

**Affiliations:** 1 Department of Virology, Research Institute for Microbial Diseases, Osaka University, Suita, Osaka 565-0871, Japan; 2 Viral Emergent Diseases Research Group (VIREM), Universidad del Valle, Cali, Colombia; 3 Department of Health Promotion Sciences, Graduate School of Medicine, Osaka University, Suita, Osaka 565-0871, Japan; 4 Regional STD Teaching, Training and Research Center, VM Medical College & Safdarjang Hospital, New Delhi, India

## Abstract

**Background:**

HIV-1 subtype C (HIV-1C) accounts for almost 50% of all HIV-1 infections worldwide and predominates in countries with the highest case-loads globally. Functional studies suggest that HIV-1C is unique in its biological properties, and there are contradicting reports about its replicative characteristics. The present study was conducted to evaluate whether the host cytokine environment modulates the *in vitro *replication capacity of HIV-1C viruses.

**Methods:**

A small subset of HIV-1C isolates showing efficient replication in peripheral blood mononuclear cells (PBMC) is described, and the association of *in vitro *replication capacity with disease progression markers and the host cytokine response was evaluated. Viruses were isolated from patient samples, and the corresponding *in vitro *growth kinetics were determined by monitoring for p24 production. Genotype, phenotype and co-receptor usage were determined for all isolates, while clinical category, CD4 cell counts and viral loads were recorded for all patients. Plasmatic concentrations of cytokines and, acute-phase response, and microbial translocation markers were determined; and the effect of cytokine treatment on *in vitro *replication rates was also measured.

**Results:**

We identified a small number of viral isolates showing high *in vitro *replication capacity in healthy-donor PBMC. HIV-1C usage of CXCR4 co-receptor was rare; therefore, it did not account for the differences in replication potential observed. There was also no correlation between the *in vitro *replication capacity of HIV-1C isolates and patients' disease status. Efficient virus growth was significantly associated with low interleukin-10 (IL-10), interleukin-22 (IL-22), and C-reactive protein (CRP) levels in plasma (p < .0001). *In vitro*, pretreatment of virus cultures with IL-10 and CRP resulted in a significant reduction of virus production, whereas IL-22, which lacks action on immune cells appears to mediate its anti-HIV effect through interaction with both IL-10 and CRP, and its own protective effect on mucosal membranes.

**Conclusions:**

These results indicate that high systemic levels of IL-10, CRP and IL-22 in HIV-1C-infected Indian patients are associated with low viral replication *in vitro*, and that the former two have direct inhibitory effects whereas the latter acts through downstream mechanisms that remain uncertain.

## Background

HIV-1 subtype C is the most prevalent HIV-1 subtype worldwide, accounting for more than 50% of HIV-1 infections worldwide in 2004 [1]. It predominates in countries with 80% of all global HIV-1 infections (sub-Saharan Africa, India) and is rapidly increasing in China and Latin America [2-5]. The reasons for the increase in HIV-1 C are not known, but may be related to host, viral, or socioeconomic factors.

Accumulating evidence suggests that HIV-1C may be unique in its spread and natural history, but the mechanistic basis for these differences remains unknown. In a West African cohort, individuals infected with HIV-1C were reported to be more likely to develop AIDS than those infected with HIV-1A [6]. In a Kenyan study, patients infected with HIV-1C showed lower CD4 cell counts and higher viral loads than patients infected with other subtypes [7]. Functional studies of HIV-1B and other subtypes have shown that progression to AIDS is associated with a selection of high-growth potential viral variants that use CXCR4 as a co-receptor [8,9]. However, HIV-1C is unique in maintaining its predominant CCR5 tropism throughout infection, which may affect its transmission and pathogenesis [10,11], and there are some contradicting reports about its replicative properties [12,13].

Subtype differences in vitality and fitness have also been reported. HIV-1C variants replicate less efficiently in macrophages and PBMC when compared to subtype B viruses, but more efficiently in Langerhans cells and in the presence of immature dendritic cells (iDCs) [14,15]. However, the utilization of diverse cellular systems and assays to measure phenotypic properties of R5 and ×4 HIV-1 viruses has led to substantial confusion in the current literature, and the true biological mechanisms underlying subtype differences in replication capacity are still poorly understood.

At the viral level, it has been suggested that an extra NF-κB binding site in the long terminal repeat may enhance gene expression, altering the transmissibility and pathogenesis of C viruses [16,17]. Other unique biological features of HIV-1C viruses include an increased responsiveness to cellular proteins and cytokines [18], low frequency of sincytium inducing (SI) viruses [19] and a number of unique subtype signatures across the viral genome [20,21].

Conversely, the influence of host factors on determining subtype differences in replication potential has received little attention in the literature. HIV-1 replication is under continuous regulation by a complex cytokine network produced by a variety of cells, and the impact of cytokines on HIV-1 replication has been amply studied in myeloid cells [22]. A number of cytokines have been reported to modulate HIV replication *in vitro*, with their effects being inhibitory (IFNα, IL-10, IL-13), stimulatory (TNF, IL-1, IL-6) or bi-functional (IL-4, IFNγ). Furthermore, a recent report showed that production of IL-10 in pregnant seropositive women helped them control HIV-1 replication and reduce the chance of transmission to the fetus [23].

Therefore, we argue that differences in the host cytokine environment affect the immune activation state, and that in turn these differences might affect the *in vitro *replication capacity of HIV viruses. Consequently, we studied whether the host cytokine environment contributes to measurable differences in replication capacity of HIV-1C viruses.

Here we identified a small number of viral isolates showing high *in vitro *replication capacity in healthy-donor PBMC. Efficient virus growth was significantly associated with a triad of low IL-10, IL-22 and CRP levels in plasma (p < .0001), and pretreatment of virus cultures with IL-10 and CRP resulted in a significant reduction of virus production *in vitro*. Additionally, systemic IL-22 levels correlated positively with CRP and IL-10, and negatively with plasmatic lipopolysaccharide (LPS), an indicator of microbial translocation from the gut; this suggests IL-22 mediates its anti-HIV effects indirectly through interactions with IL-10 and CRP, and also through its protective effect on epithelial function.

Taken together, these results indicate that a complex host environment characterized by an IL-10 dominant immunosuppressive profile that reduces immune activation, in concert with a subclinical inflammatory response of peripheral tissues mediated by IL-22 and CRP, appears to contribute to the observed low replication capacity of HIV-1C viruses in PBMC. Furthermore, both IL-10 and CRP showed direct anti-HIV action *in vitro*, whereas IL-22 given its lack of effect on immune cells appears to act through downstream mechanisms that remain poorly understood.

## Methods

### Patients and samples

Plasma and PBMC samples were obtained from 243 HIV-1-infected patients attending the Integrated Counseling and Testing Centre at Safdarjang Hospital, New Delhi, India, between February 2006 and May 2009. Of these, only the 85 subjects for whom successful viral isolation and complete biological characterization of primary isolates was possible were included in the analysis. All patients had a serologic diagnosis of HIV infection established prior to inclusion in the study, and were attending a reference center for follow-up. However, since the number of patients who were receiving HAART was very low in our sample, these cases were eliminated and only treatment-naïve patients were included in the analysis. CD4^+ ^T cells were counted by flow cytometry (Becton-Dickinson, CA). Viral load was measured by the Amplicor HIV-1 Monitor test (Hoffman-La Roche). The study was approved by the local Ethics Committee, and all patients provided their written informed consent to participate.

### Virus Isolation

HIV-1 was isolated from patients peripheral blood mononuclear cells (PBMC) when available by co-culture with phytohemagglutinin (PHA)-activated healthy-donor PBMC [24]. In the other cases, isolates were obtained by culturing 100 μl of plasma overnight with PHA-activated PBMC, as previously described [25]. Viral growth was monitored in culture supernatants every 3 days using a commercial p24 antigen kit (Zeptometrix, Buffalo, NY).

### Titration of HIV-1 isolates

The infectivity titer for each isolate was determined by measuring HIV p24 production after 7 days using an endpoint dilution assay, as described in detail elsewhere [26]. The tissue culture dose for 50% infectivity (TCID_50_) was calculated using the Spearman-Karber formula.

### Viral replication kinetics

All viral isolates that replicated to at least 100 pg/ml of p24 antigen in the initial culture were saved and further expanded by infecting new batches of PHA-stimulated PBMC from normal donors. The *in vitro *growth kinetics of the viruses studied were determined in triplicate by mono-infection of PBMC at a multiplicity of infection (MOI) of 0.001 and monitoring for p24 production over a period of 12 days post-infection (p.i.) The virus stock was diluted to obtain a MOI of 0.001 IU/PBMC, based on the TCID_50 _(IU/ml) of each viral stock and a number of 1 × 10^6 ^cells, as previously described [27]

### Phenotyping

Co-receptor usage was determined in human astroglioma U87 cell lines stably expressing CD4 and co-expressing CCR5 or CXCR4 chemokine receptors as described before [28]. Additionally, syncytial characterization was determined by the MT-2 syncytium-forming assay. For this, 1 × 10^6 ^fresh PBMCs were co-cultivated with 1 × 10^6 ^MT-2 cells, as described [29].

### Genotyping

Virus genotype was determined by conventional bulk sequence analysis of the patient samples. For this, the *pro *gene (297 bp), a p24 fragment of the *gag *gene (450 bp), and a C2-V5 fragment of the *env *gene (708 bp) of viral isolates were amplified by nested PCR and sequenced for genotype determination. Briefly, viral RNA was extracted from patient serum samples using the QIAamp Blood kit (Qiagen, Chatsworth, CA), and nested RT-PCR reactions were performed to amplify the fragments mentioned using primer sets and cycling conditions described previously [30,31]. As a positive control, we used an infectious molecular clone of Indian HIV-1C, Indie-C1 [32]. PCR products were directly sequenced using a Big Dye Terminator, version 1.1 (Applied Biosystems), in accordance with the manufacturer's instructions. Sequences were trimmed manually and aligned with sequences representative of the HIV-1 group M subtypes available in the Los Alamos database using CLUSTALX [33].

### Determination of cytokines, acute-phase response and microbial translocation markers

Plasmatic concentrations -in pg/ml- of tumor necrosis factor-alpha (TNF-α), interferon-gamma (IFNγ), interleukin (IL)-1, IL-4, IL-6, IL-10, IL-17, IL-22, and C-reactive protein (CRP) -in μg/ml- in patients' samples and HIV-negative controls were determined quantitatively using commercially available colorimetric immunoassays (Quantikine Human Immunoassay kits) and carried out as recommended by the manufacturer (R&D Systems, Minneapolis, MN). Plasma LPS levels were determined as described elsewhere [34], using the *Limulus *amebocyte lysate assay (LAL) according to manufacturer's instructions (Lonza, Walkersville, MD).

### Cytokine treatment of viral cultures

Healthy-donor PBMCs were pre-incubated for 1 hour at 37°C with several treatment schemes of recombinant human IL-6, IL-10, CRP and anti-IL-10 mAb, followed by incubation with 2 representative R/H phenotype HIV-1C primary isolates and the infectious molecular clone Indie-C1, at a MOI of 0.1. After incubation for 2 h, the cells were extensively washed and cultured for 12 additional days. All treatments were added at concentrations just high enough to elicit a maximum response, as measured by the ability to bind Fcγ RIIa in case of CRP, and in a cell proliferation assay using a factor-dependent cell line in case of the interleukins [35,36]. The anti-IL-10 antibody was added at the Neutralization Dose_50 _(ND_50_). Differences in virus growth compared with untreated controls were evaluated by monitoring p24 production.

### Statistical analysis

Comparisons between groups were performed using the Pearson chi-square or Fisher's exact test for categorical variables. The distribution of continuous variables (viral load, CD4 cell count, etc.) was compared between patients with different viral replication phenotypes using non-parametric methods (Mann-Whitney U test). The non-parametric Spearman rank correlation test was used to analyze the correlation between the *in vitro *replication capacity and disease progression, cytokine production profile and disease progression, as well as the correlation between systemic IL-22 levels and the acute-phase and microbial translocation markers. Use of these non-parametric statistics was required for the analysis of observations that were not normally distributed. Fisher's *z *transformation was used to calculate confidence intervals to test the statistical significance of the correlation coefficients (i.e., to determine the degree of confidence that the true value of the correlation in the population is contained within these intervals). Statistical significance was defined as *p *< .05 values.

## Results

### Epidemiological and clinical characteristics of patients

Table 1 summarizes the epidemiologic, clinical, laboratory and virological data of the patients. The study sample (n = 85) comprised 53 men and 32 women with an age distribution ranging from 10 to 55 years (mean, 33.37 years). Infection with HIV-1 occurred primarily through heterosexual contact (82.4%), but with a clear distinction according to gender. Transmission in men was associated with extramarital activities, while women acquired the virus mainly through their infected spouses, and this difference was statistically significant (*p *< .001). The mean viral load in plasma was high (230,767 HIV-1 RNA copies/ml) consistent with advanced HIV-1 disease, but, while the mean CD4^+ ^T-cell count was low (255.6 cells/mm^3^), it never fell below AIDS levels in almost half of the patients.

**Table 1:** Characteristics of 85 Indian HIV-infected patients with R/H or S/L phenotype HIV-1C isolates

	S/L^† ^group (n = 72)	R/H^‡ ^group (n = 13)	Total (n = 85)	*p*
	number (%) *	number (%) *	number (%) *	
**EpidemiologicalData**				
Sex				0.758
Male	44 (61.1)	9 (69.2)	53 (62.4)	
Female	28 (38.9)	4 (30.8)	32 (37.6)	
Age (mean years ± SEM^§^)	33.7 ± 1.1	31.4 ± 1.5	33.4 ± 0.9	0.426
Transmission route				0.184
Heterosexual: extramarital	31 (43.1)	6 (46.2)	37 (43.5)	
Heterosexual: infected-partner	31 (43.1)	2 (15.4)	33 (38.8)	
Others	10 (13.9)	5 (38.5)	15 (17.6)	
**Clinical Data**				
Common clinical findings				
Fever of Unknown Origin (FUO)	27 (37.5)	6 (46.2)	33 (38.8)	0.554
Acute febrile syndrome	23 (31.9)	8 (61.5)	31 (36.5)	0.060
Asymptomatic	17 (23.6)	3 (23.1)	20 (23.5)	1.000
Diarrea	18 (25.0)	2 (15.4)	20 (23.5)	0.724
Pulmonary TBc	17 (23.6)	2 (15.4)	19 (22.4)	0.723
Others	24 (33.3)	6 (46.2)	30 (35.3)	0.529
CDC Clinical category^1^				0.342
A	27 (37.5)	4 (30.8)	31 (36.5)	
B	19 (26.4)	6 (46.2)	25 (29.4)	
C	26 (36.1)	3 (23.1)	29 (34.1)	
CDC CD4 category^2^				0.063
1	8(11.1)	2 (15.4)	10 (11.8)	
2	30(41.7)	1 (7.7)	31 (36.5)	
3	34(47.2)	10(76.9)	44 (51.8)	
Combined CDC AIDS Category^3^	41 (56.9)	10(76.9)	51 (60.0)	0.227
**Virological Data**				
Genotype				1.000
HIV-1C	72(100)	13(100)	85(100)	
Phenotype				1.000
R5	68 (94.4)	13(100)	81 (95.3)	
×4	4 (5.6)	0 (0)	4 (4.7)	
p24 Ag titer (pg/ml; mean ± SEM^§^)	189.0 ± 5.8	1745.7 ± 64.9	427.1 ± 62.1	<0.001^¶^

^†^S/L, Slow low viral growth phenotype; ^‡^R/H, Rapid high viral growth phenotype.

* The percentages were calculated by dividing the number of observations with a given characteristic by the total number of subjects in each category

^1 ^CDC Classification System & Expanded AIDS Surveillance Definition for Adolescents and Adults. Based on 3 clinical categories; category C includes the clinical conditions listed in the AIDS surveillance case definition.

^2^CDC Classification System & Expanded AIDS Surveillance Definition for Adolescents and Adults. System of 3 ranges of CD4 counts based on the lowest documented measure; category 3 <200/μL CD4 cells.

^3^The definition of AIDS includes all HIV-infected individuals with CD4 counts of <200 cells/μL as well as those with the clinical conditions listed in the AIDS surveillance case definition (categories A3, B3, and C1-C3).

^¶^Overall *p*-value less than 0.001. ^§^SEM, standard error of the mean.

Comparisons between groups were performed using the Pearson chi-square or Fisher's exact test for categorical variables. The Mann-Whitney U test was used for continuous data.

### A small subset of viral isolates that replicate efficiently in PBMC

**Figure 1:**
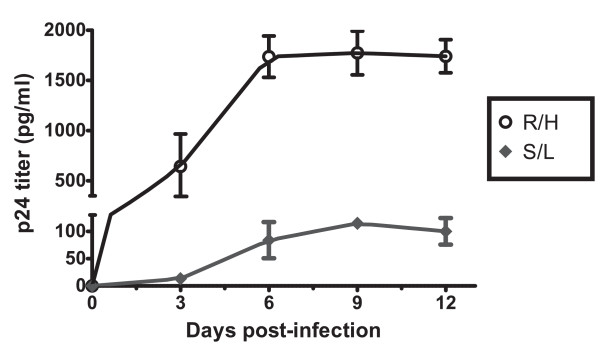
**Viral replication kinetics of HIV-1C isolates on PHA-activated healthy-donor PBMC**. Virus replication was monitored by measuring the amounts of p24 Gag protein produced in the culture supernatants every three days. The values given are mean ± SD of p24 antigen (pg/ml) of either R/H isolates (open circle) or S/L isolates (filled diamond). The data are representative of the results from three independent experiments.

Direct co-culture of patient PBMC/plasma with healthy-donor cells was used for viral isolation, as described in Methods. Of the 85 co-cultures which resulted in successful viral isolation, most (75%) became positive in ≤ 6 days and reached peaks of p24 production by day 9, although replication patterns varied widely. As shown in Fig. 1, thirteen isolates had a suggestive "rapid/high" (R/H) replication phenotype, replicating quickly and to high titers in primary and secondary expansion cultures, reaching high levels of p24 production (mean, 1745.7 pg/ml). The other 72 isolates did not produce high levels of virus in primary or secondary expansion cultures (mean, 188.9 pg/ml), showing a suggestive "slow/low" (S/L) replication pattern [37,38].

### Biological characterization of viral isolates

A frequently cited reason for enhanced cytopathicity and more vigorous viral replication is the development of viral variants that use CXCR4 as co-receptor [8]. Therefore, to further characterize the biology of our HIV-1C isolates, syncytial phenotype and co-receptor usage were determined by means of the MT-2 syncytial assay and the U87.CD4 cell assay, respectively. As shown in Table 1, all but four of the primary isolates used in this study (n = 85) replicated more efficiently in U87. CD4 cells expressing CCR5 than in CXCR4-expressing cells, indicating that they are R5 tropic viruses. The fold difference of CCR5 over CXCR4 growth was nearly 100-fold, and the HIV p24 values in U87.CD4.CXCR4 cells were mostly below 150 pg/ml. Consistent with CCR5 co-receptor usage, isolates were found to be non-syncytium inducing (NSI) due to their inability to form syncytia in MT2 cells. More importantly, the only four isolates with preferential ×4 tropism were all S/L viruses. These results indicate that the presence of R/H isolates in our study is independent of ×4 co-receptor usage. Additionally, all HIV-1 isolates were subtyped in the C2-V5 region of the *env *gene by direct sequencing and were shown to belong to HIV-1C, the subtype predominant in 99% of infections in India [39]. Thirty isolates were also subtyped in the *pro *and *gag *genomic regions and shown to belong to subtype C, suggesting that these isolates were unlikely to be intersubtype recombinants, although this cannot be excluded.

### Efficient replication does not correlate with disease progression

**Figure 2:**
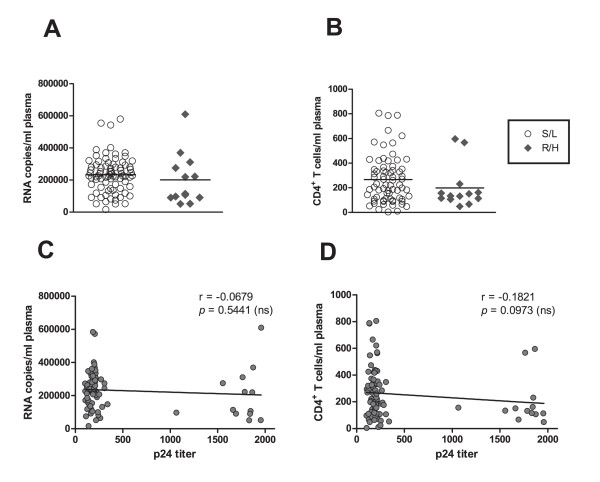
**Viral load and CD4^+ ^T-cell count in HIV-1C-infected Indian patients**. The indicated parameters were evaluated in 85 HIV-1C-infected individuals included in this study and sorted according to viral growth phenotype. (A) Viral load (HIV RNA copies/ml plasma) in patients with either R/H (open circle) or S/L viral isolates (filled diamond). (B) CD4^+ ^T-cell counts (cells/mm^3^) in the same groups of patients. (C) Lack of correlation between viral load (HIV RNA copies/ml plasma) and *in vitro *replication (p24, pg/ml) in the same sample. (D) Lack of correlation between CD4^+ ^T-cell counts (cells/mm^3^) and *in vitro *replication (p24, pg/ml). For panels C and D, r = Spearman correlation coefficient; *p*-value (two tailed);*(ns) *indicates that the *p*-value of the correlation coefficient was more than .05 (not significant). The mean values of the measurements obtained from two independent experiments are shown (n = 85).

Since viral strains with high-growth potential are selected in late-stage disease [40], we analyzed the relationship between markers of disease progression and viral replication. We categorized disease progression in our 85 HIV-infected patients according to viral loads [41] as well as the 1993 CDC Classification System & Expanded AIDS Surveillance Definition for Adolescents and Adults [42], which classifies patients on the basis of clinical conditions associated with HIV infection and CD4^+ ^T- lymphocyte counts. Clinical status and the CDC classification results are summarized in Table 1, whereas analysis of viral load and CD4^+ ^T-cell count is shown in Fig. 2A and 2B, respectively. No significant differences were found between R/H and S/L isolates when clinical category (*p *= .342), virus load (*p *= .455) or CD4^+ ^T cell category (*p *= .063) were compared, suggesting similar disease status for both groups of subjects. Additionally, correlation analysis further showed the lack of association between replication and viral load or CD4^+ ^cell counts as shown in Fig. 2C and 2D, respectively.

### Replication capacity of isolates is negatively associated with patient plasmatic IL-10, IL-22 and CRP levels

**Figure 3:**
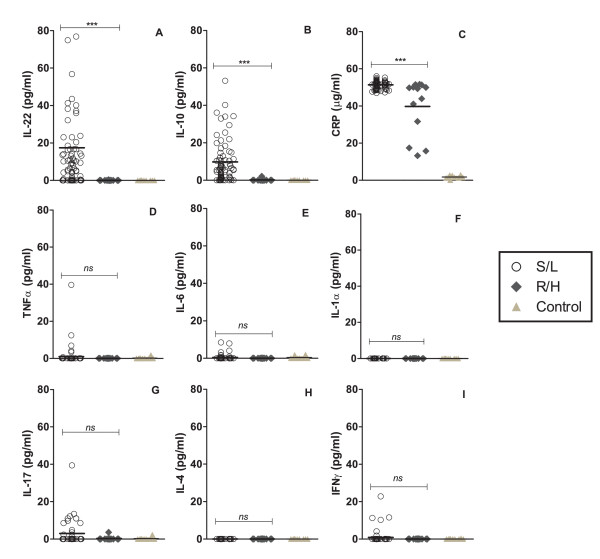
**Cytokine profile of HIV-1C infected Indian patients**. The indicated cytokines, along with the inflammatory marker CRP were evaluated by ELISA in plasma collected from HIV-1C-infected patients (n = 85) harboring either R/H (filled diamond) or S/L (open circle) growth phenotype viruses, or in 10 HIV-uninfected healthy controls (filled triangle). The mean systemic values of each cytokine (pg/ml) and CRP (μg/ml) are compared in the figure for R/H and S/L viral phenotype groups. TNF-α, tumor necrosis factor-alfa; IFNγ, interferon-gamma; IL-1α, interleukin-1 α; IL-4, interleukin-4; IL-6, interleukin-6; IL-10, interleukin-10; IL-17, interleukin 17; IL-22, interleukin-22; CRP, C-reactive protein. Extreme outlier data points (IL-22) are not depicted for better visualization of results, but included into all calculations. *** = statistical difference of the medians, *p *< 0.001. The mean values of the measurements obtained from two independent experiments are shown.

The *in vitro *replication capacity of the viral isolates was determined by monitoring p24 antigen production; and according to their p24 production profiles, two groups of isolates -R/H and S/L- were observed, as already noted. When the cytokine responses were compared between R/H and S/L isolates, we found a strong negative association between the plasmatic levels of IL-22 (*p *= .00004) and IL-10 (*p *= .0000007) and the *in vitro *replication kinetics of HIV-1C primary isolates, as shown in Fig. 3A and 3B, respectively (n = 85). No statistical differences in plasmatic levels of TNF-α, IFNγ, IL-4, IL-6, IL-1α, and IL-17 were observed between the two groups, Fig. 3D through 3I. For comparison, the plasmatic concentrations of all cytokines in 10 HIV-uninfected healthy control subjects are shown, but as expected most measurements fell below the detection limit, given that the transient and paracrine character of cytokines limits its detection in the absence of systemic pathology [43].

Importantly, IL-22 does not target immune cells [44], therefore it has no effect on the immune response. However, the literature suggests that the anti-HIV activity of this cytokine could be mediated by its downstream acute-phase products [45]. Subsequently, we measured the plasmatic levels of the pentraxin CRP (Fig. 3C), and found that it was also inversely associated with viral replication (*p *= .0003). Nevertheless, although CRP levels were significantly different between both R/H- and S/L-harboring patients (mean, 39.75 and 51.35 μg/ml, respectively), the concentrations of plasmatic CRP found were well under 10 mg/l, and thus do not reflect clinically significant inflammatory states, but rather a subclinical response [46]. CRP levels in HIV-uninfected controls (mean 1.75 μg/ml) were >20-fold lower than the levels detected in our patient population, in accordance with the low concentrations of CRP reported to circulate in the absence of acute infective or inflammatory episodes [47] and the manufacturer's calibration data.

### No correlation between production of IL-10, IL-22 and CRP and the disease status of HIV-1C-infected Indian patients

Next we sought to lend support to the association between cytokine production and the rate of viral replication in our *in vitro *model with *in vivo *data monitoring disease progression. It is expected that a combination of viral and host factors will determine how fast can HIV replicate or overcome the immune response *in vivo*, thus dictating the rate of progression to AIDS in untreated patients. Therefore we calculated the Spearman correlation coefficients for plasmatic levels of IL-10, IL-22 and CRP and disease progression as defined by patients' viral load, CD4 cell counts, and AIDS-defining conditions. For comparison, other clinical syndromes not associated with late-stage disease but highly prevalent in our study sample were also evaluated. Additionally, the 95% confidence intervals for the correlations were calculated to test the statistical significance of the correlation coefficients as described in Methods. Table 2 shows the results of these correlation analyses. The only significant correlation found, as seen in the table, was between IL-22 titers and the presence of idiopatic chronic diarrhea, a common finding of clinically latent or mildly symptomatic disease. The relevance of this finding will be discussed later in the paper. Meanwhile, we found no correlation between the plasmatic titers of IL-10, IL-22 and CRP, and the disease progression markers studied. However, this was not entirely surprising given the polygenic and multifactorial nature of the disease, and the difficulties in isolating the effect of individual genes/proteins amongst unknown environmental and genetic factors pertaining to both the human host and the virus [48]. Even though the role in suppressing HIV replication of several host factors (cytokines, β-chemokines, chemokine receptors, APOBEC3G, TRIM5α, TSG101, etc.) has been amply documented in *in vitro/ex vivo *models, consistent associations with progression to AIDS have not been observed in genetically diverse cohorts of patients [49-53]. Allelic variations of each host factor, genetic differences between ethnicities and other factors have also confounded the characterization of significant associations. Additionally, one would expect that if the cytokine activity results in slower HIV-1 replication *in vitro*, presumably this would translate in lower viral loads *in vivo*; however, we must consider that several confounders affect this outcome as well. Subtype differences for one, as patients infected with HIV-1C have shown lower CD4 cell counts and higher viral loads than patients infected with other subtypes in previous reports [7], are in agreement with our results. Also several studies support a role for co-infecting pathogens in HIV disease. For example, malaria episodes and TB infection result in increase HIV viral load [54], and the latter has been shown to accelerate the course of HIV [55]. An argument for TB is particularly relevant in India, where HIV/TB co-infection rates are as high as 47% in Delhi [56], and it has been shown to represent the etiology of 63% of FUO cases [57,58]. Furthermore, viral load does not predict *in vitro *infectivity, since current methods routinely used for viral load measurements determine the amount of genomic HIV-1 RNA copy numbers, but cannot distinguish between infectious and noninfectious or neutralized particles in plasma [59]. We recognize that the lack of potential mechanisms to explain the effect of these cytokine phenotypes *in vivo *warrants the need for additional studies; nevertheless, characterizing the small contribution of single effects and developing multifactorial models that take into account the combined effects of multiple variants is out of the scope of the present work.

**Table 2:** Lack of correlation between IL-10, IL-22 and CRP production and disease progression

	IL-10		IL-22		CRP	
	*r* (95% C.I.)^§^	*p*	*r* (95% C.I.)	*p*	*r* (95% C.I.)	*p*
Viral load	0.213 (-0.004 - 0.411)	ns^¶^	0.118 (-0.102 - 0.327)	ns	0.010 (-0.207 - 0.226)	ns
CD4 count	-0.146 (-0.349 - 0.071)	ns	-0.073 (-0.283 - 0.144)	ns	0.018 (-0.197 - 0.231)	ns
AIDS-defining illness^a^						
Pulmonary TB^†^	0.041 (-0.174 - 0.252)	ns	-0.005 (-0.218 - 0.207)	ns	-0.054 (-0.264 - 0.161)	ns
Cryptosporidiosis^+^; isosporiasis	0.165 (-0.05 - 0.364)	ns	0.178 (-0.037 - 0.377)	ns	0.085 (-0.13 - 0.293)	ns
Wasting syndrome	0.122 (-0.094 - 0.326)	ns	0.101 (-0.115 - 0.307)	ns	0.161 (-0.054 - 0.362)	ns
Lymphoma	-0.089 (-0.297 - 0.126)	ns	0.096 (-0.12 - 0.303)	ns	-0.022 (-0.234 - 0.192)	ns
Other clinical Sx^‡^						
F.U.O.^⋇^	0.158 (-0.057 - 0.359)	ns	0.098 (-0.118 - 0.305)	ns	0.039 (-0.176 - 0.250)	ns
Chronic Diarrhea^o^	0.172 (-0.043 - 0.371)	ns	0.388 (0.191 - 0.555)	<0.001	0.168 -0.047 - 0.231	ns
Constitutional symptoms	0.015 (-0.199 - 0.227)	ns	0.033 (-0.181 - 0.244)	ns	-0.093 (-0.3 - 0.123)	ns

^§ ^*r*, Spearman correlation coefficient; 95% C.I., 95% confidence interval.

^¶ ^ns, not significant (overall *p*-value of more than 0.05).

^a^Clinical conditions listed in the AIDS surveillance case definition; only the most frequent in our study sample are shown.

^† ^Pulmonary TB, pulmonary tuberculosis.

^+^Chronic, intestinal (> 1 month)

^‡ ^Other clinical syndromes, non AIDS defining.

^⋇ ^F.U.O., Fever of Unknown origin.

^o^Idiopatic diarrhea, > 1 month.

### Effect of recombinant cytokine treatment on HIV-1C replication kinetics in vitro

**Figure 4:**
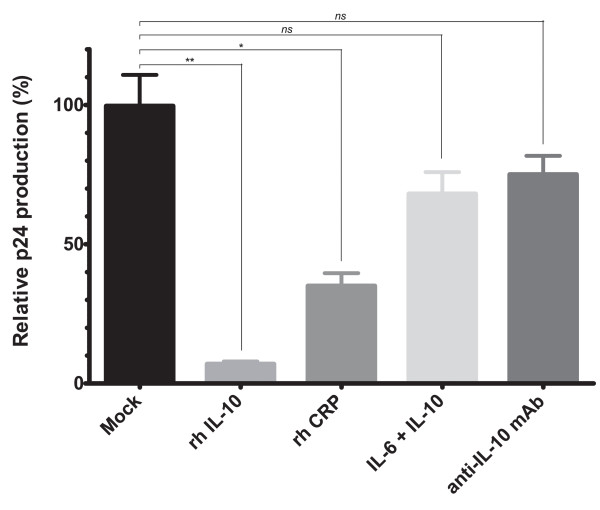
**IL-10 and CRP pre-treatment of viral expansion cultures downregulates HIV-1C replication *in vitro***. PHA-activated healthy-donor PBMCs were pre-incubated with recombinant human IL-6 (2.5 ng/ml), IL-10 (5 ng/ml) or CRP (1 μg/ml), for 1 h before infection with a representative R/H phenotype HIV-1C primary isolate, and HIV-1 p24 levels were determined by ELISA after 7 days of culture. Treatments schemes are shown as follows: (1) Mock; (2) recombinant human (rh) IL-10; (3) rh CRP; (4) IL-6+IL-10; anti-IL-10 mAb+IL-10. The data are representative of the results from two independent experiments. Results are shown as mean ± SD of relative p24 antigen production expressed as percent of the mean p24 titer measured in the Mock-treated cultures. * = statistical difference of the medians, *p *< 0.05; ** = statistical difference of the medians, *p *< 0.01; *(ns) *indicates that the *p*-value of the correlation coefficient was more than .05 (not significant).

To further confirm the role of IL-10 and CRP in regulating *in vitro *replication of HIV-1C, we tested the effect on virus growth of pretreatment of virus expansion cultures with maximum-response doses of CRP and recombinant human IL-10 either alone or in combination with IL-6 and anti-IL mAb. IL-6 treatment was used to stimulate virus production, and the blocking effect of neutralizing doses of anti-IL-10 mAb was also evaluated. The p24 production in all treatment schemes was compared with untreated controls. As shown in Fig. 4, addition of recombinant IL-10 (5 ng/ml) to the media of virus expansion cultures of representative R/H phenotype HIV-1C isolates showed a >10-fold reduction in viral titers *in vitro*, which was statistically significant (*p *= .009). This effect was partially abolished by co-incubation with the stimulatory cytokine IL-6 (2.5 ng/ml). This is in agreement with reports which suggest that the anti-HIV activity of IL-10 derives from its block on the production of inflammatory cytokines [60]. Likewise, a concentration equal to the ND_50 _dose of anti-IL10 also restricted the negative effect of IL-10 on replication, further highlighting the role of this cytokine in controlling viral growth. Additionally, CRP treatment (1 μg/ml) also showed a lesser, yet significant inhibition of viral replication (*p *= .021) in accordance with previous reports that show that acute-phase proteins can have anti-HIV activity [61].

### Systemic IL-22 levels positively correlate with plasmatic CRP and IL-10, and negatively with plasmatic LPS

**Figure 5:**
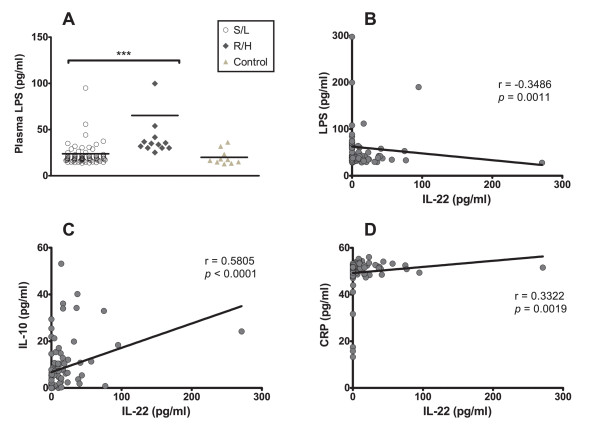
**Correlation between systemic IL-22 and plasmatic CRP, LPS and IL-10 levels**. (A) Systemic LPS levels were evaluated by the LAL assay, in plasma collected from HIV-1C-infected patients (n = 85) harboring either R/H (filled diamond) or S/L (open circle) growth phenotype viruses, or in 10 HIV-uninfected healthy controls (filled triangle). The mean plasmatic LPS values (pg/ml) are compared in the figure for R/H and S/L viral phenotype groups. Extreme outlier data points are not depicted for better visualization of results, but included into all calculations. *** = statistical difference of the medians, *p *< 0.001. (B) Correlation between plasma LPS (pg/ml) and plasma IL-22 levels (pg/ml). (C) Correlation between plasma IL-10 (pg/ml) and plasma IL-22 levels (pg/ml). (D) Correlation between plasma CRP (pg/ml) and plasma IL-22 levels (pg/ml) in our study sample (n = 85). In all panels, r = Spearman correlation coefficient; *p*-value (two tailed);*p*-values of the correlation coefficient of less than .05 were considered significant. The mean values of the measurements obtained from two independent experiments are shown.

Since IL-22 does not act on immune cells given that lymphocytes lack IL-22 receptors [62], it was not possible to evaluate its effect on viral replication using the same *in vitro *model that was applied for IL-10 and CRP; and therefore an alternative strategy was sought. IL-22 is bi-functional with both pro-inflammatory and protective effects on tissues depending on the inflammatory context. As previously shown in Table 2, inspection of the relationship between the cytokine response and patient clinical manifestations revealed that IL-22 correlated with chronic idiopatic diarrhea (*p *< .001), which is interesting given that IL-22 appears to play an important role in maintaining the integrity of the epithelium in animal models of intestinal infection [63]. It has been shown that higher circulating levels of bacterial byproducts (as a result of increased translocation into the bloodstream) are associated with increased levels of immune activation, which in turn correlates with increased HIV-1 disease progression [64]. Subsequently we measured the plasmatic levels of LPS, a component of Gram-negative bacteria that acts as a marker of microbial translocation from the gut, in all patients (n = 85), and in 10 HIV-uninfected healthy individuals for comparison. As shown in Fig. 5A, we found statistically significant lower plasma concentrations of LPS in patients harboring S/L when compared to their R/H counterparts (p < .0001), suggesting lower levels of microbial translocation in the S/L group. LPS levels in healthy controls were in the lower range of titers reported in previous studies [34,65]. More importantly, IL-22 showed a significant negative correlation with plasmatic LPS (*p *= .001), as shown in Fig 5B, suggesting a protective role on the epithelial barrier. Taken together, these results support the hypothesis that the actions in mucosal host defense and epithelial barrier protection described for IL-22 [66] are also required to exert its anti-HIV actions, although its exact mechanisms of action are presently unknown.

On the other hand, the plasmatic levels of both IL-22 and IL-10 were also intrinsically correlated with each other (Fig. 5C), and this association was statistically significant (*p *< .001), as was the correlation between IL-22 and CRP (*p *= .002) as shown in Fig. 5D, but not between IL-10 and CRP (*p *= .883, not shown). As demonstrated before, both IL-10 and CRP significantly inhibit HIV-1C replication *in vitro*, so this result suggests that IL-22 might also mediate its anti-HIV effects indirectly through association with IL-10 and CRP, although the exact mechanisms remain unresolved.

## Discussion

Reports of a reduced replication capacity of HIV-1C in primary blood cells (CD4^+ ^T cells and PBMC) have led some to suggest HIV-1C has low virulence [67]. However, reduced fitness in a reconstituted *in vitro *assay does not necessarily imply that the corresponding virus will be less fit in a human host, since in the *in vivo *environment, a complex array of host factors can influence HIV-1 replication. Therefore, we believe that it is impossible to discuss differences in the viral replication of HIV-1C isolates without reference to the multidimensional environment of the host from which they were obtained.

The role of immunologic host factors has been widely discussed in recent literature. HIV disease is described as a disease of immune activation, yet the causes of immune activation are not fully understood [68]. Conversely, lack of immune activation has been shown to protect SIV-infected sooty mangabeys (SM) and African green monkeys from progressing to AIDS [69]. It has also been shown that production of cytokines (IL-2, TNFα, and IFNγ), particularly from dendritic cells, is quantitatively reduced in SM compared to rhesus macaques and that expression of IFNα is absent in SM [70]. This modulation in response sets the stage for nonpathogenic SIV-SM coexistence, while the same triggers in rhesus macaques and humans result in generalized activation of the immune system and immunopathogenesis.

Here we described 13 viral isolates showing high replication capacity in PBMC, a target in which HIV-1C is reported to have low fitness. HIV-1C usage of the CXCR4 co-receptor was rare and did not account for the differences in replication between R/H and S/L viruses. There was also no correlation between the *in vitro *replication kinetics of the isolates and all markers of disease progression, but CD4 cell counts were low and viral loads were high for both groups and in all stages of disease, in accordance with previous reports [7].

Most importantly, the data presented here show that the ability of HIV-1C viruses to replicate efficiently in activated healthy-donor PBMC is negatively associated with plasmatic levels of IL-10 and IL-22, as well as the inflammatory marker CRP in the corresponding patients, and that treatment of viral expansion cultures with recombinant IL-10, and CRP inhibits replication of HIV-1C viruses *in vitro*.

This finding is consistent with previous reports that showed the stimulation of monocyte-derived macrophages (MDM) with IL-10 significantly reduces HIV-1 production in those cells [22], and this inhibition is associated with the ability of IL-10 to downregulate production of IL-6 and TNFα [60]. Additional support for the role of IL-10 on modulation of HIV-1 replication comes from recent studies showing that IL-10-secreting T cells downregulate HIV replication in pregnant women [23] and elderly patients [71].

Conversely, CRP is an acute-phase reactant rapidly synthesized in response to tissue injury, and it is widely used as an unspecific indicator of an acute inflammatory response. Its biological effects are similar to those of immunoglobulins, precipitating, acting as a primitive opsonin to bind to macrophages, and fixing complement, but it lacks immunomodifying actions [72]. Furthermore, an elevation in the baseline concentration of CRP below 10 mg/l, like the one observed in this study, is termed "low-grade inflammation" and considered to reflect housekeeping responses to distressed or injured cells, rather than clinically significant inflammatory conditions.

Perhaps the most interesting implication of this study concerns the nature of the correlation between IL-22-in association with IL-10 and CRP- and viral replication. IL-22 is an important Th17 T-cell-associated cytokine that mediates tissue responses during inflammation, but it also has a role in the host response to infectious diseases [63]. Importantly, the IL-22 receptor (IL-22R) is highly expressed within tissues, primarily in epithelial cells, but it is absent on immune cells; therefore, its activation has no direct effect on the immune response [73]. Even though little is known about the interaction between IL-22 and IL10, at least one report suggests that IL-22 induces IL-10 mRNA and protein production in a colon epithelial cell line [74]. Therefore, one possible explanation for the anti-HIV effects of IL-22 could be the upregulation of IL-10 production, but, given the lack of systematic studies on whether IL-22 modulates IL-10 effects or vice versa, this explanation remains speculative.

Additionally, IL-22 has been shown to strongly induce production of acute-phase proteins in the liver [74,75]; so it could be suggested that the minor elevations in the baseline concentrations of CRP that we observed could be explained by the inflammatory role of IL-22. In this study, increased levels of IL-22 were reflected in a specific increase in plasmatic levels of CRP, confirming an association between the biological functions of both molecules. Of note, the secretion of CRP by hepatocytes and epithelial cells is induced not only by IL-22, but also by several other pro-inflammatory cytokines (IL-1, IL-6, IL-17, TNF-α). However, as the immunoassay results did not reveal high levels of the latter cytokines, it is likely that the observed CRP levels result principally from the action of IL-22.

Importantly, acute-phase proteins are reported to have strong anti-infectious activity on some HIV-1 isolates [61], and one of the mechanisms potentially implicated is a downregulation of HIV-1 co-receptors. CRP has been particularly implicated in a strong downregulation of CCR5 [76], the main co-receptor used by HIV-1C viruses. In our case this evidence is particularly relevant, since IL-10, the other cytokine implicated in our study does not affect the surface expression of CCR5 [77]. Therefore, another possible scenario would be that the anti-HIV activity of IL-22 is indirectly mediated through its downstream acute-phase products. Additional support for this hypothesis comes from a previous study that showed that IL-22 is upregulated in repeatedly exposed but uninfected individuals, concomitantly with the acute-phase product serum amyloid protein-A, which inhibited HIV infection of DCs *in vitro *[45].

Finally, the role of IL-22 on mediating protective host responses against extracellular pathogens may contribute to its anti-HIV actions. As mentioned before, IL-22 appears to play an important role in maintaining the integrity of the epithelium. In murine models of intestinal infection with *Citrobacter rodentium*, there is greater intestinal damage in the absence of IL-22, leading to greater levels of systemic bacteria [63]. Indirect support for this interpretation comes from recent studies showing that increased translocation of intestinal bacterial byproducts into the circulation, is found in advanced HIV-1 infection and is associated with an increase in immune activation [34,78]. It is important to note that in this study we found a negative correlation between systemic levels of IL-22 and plasmatic LPS, a marker of microbial translocation. These results suggest that protection of the intestinal epithelial barrier and enhancement of wound repair conferred by IL-22 production are also critical elements of its anti-HIV effects. This interpretation is partially supported by previous work showing that plasma LPS levels decrease after initiating anti-retroviral therapy (ART), which suggests an intrinsic relationship between reduced viral replication and integrity of the intestinal mucosa, although the exact molecular mechanisms involved remain unresolved.

The findings presented here support the role of IL-10 in controlling viral replication, and provide a starting point for further investigation of the influence of IL-22 and CRP on HIV replication. There are obvious limitations in our work, as it would have been desirable to evaluate directly if there were any differences in cytokine production between the PBMC of S/L and R/H subjects, or differences in the surface expression of CCR5 between those groups, since this data would better support some of the findings and conclusions presented here. Unfortunately, due to problems in the practical implementation of patient collection and low return rates, we could not address these questions. Despite these reservations, we feel our preliminary findings suggest an interesting avenue for research on HIV-1C pathogenesis, and some of the questions raised warrant further investigation.

## Conclusions

In summary, the present study supports the hypothesis that high systemic levels of IL-10, IL-22 and CRP in HIV-1C-infected Indian patients correlate with a reduced replication *in vitro*. This triad sets up an environment characterized by an IL-10-mediated immunosuppressive profile that reduces immune activation, in concert with a subclinical inflammatory response of peripheral tissues mediated by IL-22 and CRP. Additionally, both IL-10 and CRP demonstrate a direct inhibitory effect on the *in vitro *replication of HIV-1C viruses, whereas IL-22 appears to exert its effect through downstream mechanisms that are still not well understood, but that include both its inflammatory and protective actions. The anti-HIV action of IL-10 has been widely documented, but to the best of our knowledge this is the first study reporting preliminary evidence of an association of IL-22 and CRP with low viral replication. However, the molecular and cellular mechanisms underlying the difference in cytokine production between patients harboring R/H or S/L viruses, the logistics of cooperation between IL-22, IL-10 and CRP, as well as the correlation between intestinal protection conferred by IL-22 and control of viral replication, remain elusive and will be the subject of future investigation. We hope that in the future, a better understanding of the mechanisms responsible for the inhibition of viral replication exerted by IL-10, IL-22 and CRP will provide advances in our understanding of the pathogenesis of AIDS and eventually translate into future therapeutic strategies to be used in concert with ART in HIV-infected individuals.

## Competing interests

The authors declare that they have no competing interests.

## Authors' contributions

JFA did most of the experimental work and contributed with KI to the design of the study, interpretation of the data and writing of the manuscript. RN performed the statistical analyses and contributed to the interpretation of the data and drafting of the paper. MB assisted in patient and data collection and contributed to virus isolation and replication kinetics analyses, as well as critical review of the manuscript.
